# A Newly Discovered Epidemic Area of *Echinococcus multilocularis* in West Gansu Province in China

**DOI:** 10.1371/journal.pone.0132731

**Published:** 2015-07-17

**Authors:** Jian Han, Genshu Bao, Duoqiang Zhang, Pengcheng Gao, Tinjun Wu, Philip Craig, Patrick Giraudoux, Xiao Chen, Qi Xin, Lili He, Gen Chen, Tao Jing

**Affiliations:** 1 Department of Pathogenic Biology, School of Basic Medical Sciences, Lanzhou University, Lanzhou, China; 2 The Research Institute of Biomedical Nanotechnology, School of Basic Medical Sciences, Lanzhou University, Lanzhou, China; 3 Department of Hepatobiliary Surgery, Ningxia People’s Hospital, Yinchuan, China; 4 Department of General Surgery, Zhangye People’s Hospital, Zhangye, China; 5 Center for Disease Control of Minle County, Minle, China; 6 Cestode Zoonoses Research Group, School of Environment and Life Sciences, University of Salford, Manchester, United Kingdom; 7 Chrono-environment lab, University of Franche-Comté, Besançon, France; 8 Department of General Surgery, Lanzhou University Second Hospital, Lanzhou, China; Oxford University, UNITED KINGDOM

## Abstract

Alveolar echinococcosis (AE) is a lethal parasitic disease. In Gansu Province of China, all AE cases reported in literature were from Zhang and Min Counties, the southern part of the province. Here, we report the discovery of nine AE cases and one cystic echinococcosis (CE) case from Nanfeng Town of Minle County, in the middle of Hexi Corridor in west Gansu Province. The diagnosis of these cases were confirmed by serology, histopathology, computed tomography, B-ultrasound, immunohistochemistry method, DNA polymerase chain reaction and sequencing analysis. Because eight of nine AE cases came from First *Zhanglianzhuang* (FZLZ) village, we conducted preliminary epidemiological analyses of 730 persons on domestic water, community and ecology such as 356 dogs’ faeces of FZLZ, in comparison with those of other five villages surrounding FZLZ. Our studies indicate that Nanfeng Town of Minle County is a newly discovered focus of AE in China as a CE and AE co-epidemic area. Further research of *Echinococcus multilocularis* transmission pattern in the area should be carried for prevention of this parasitic disease.

## Introduction

Echinococcosis is a worldwide, serious zoonosis caused by the larval stages of tapeworms of the genus *Echinococcus*. Four species of *Echinococcus* (*E*. *granulosus*, *E*. *multilocularis*, *E*. *vogeli*, *E*. *oligarthrus*) are known to be pathogenic and of public health concern [1,2].

Alveolar echinococcosis (AE), a serious disease in humans and animals, is caused by *E*. *multilocularis*, and was found many years ago [3]. The metacestode larvae of *E*. *multilocularis* are an infiltrating structure consisting of many small vesicles embedded in the stroma of connective tissue. The larvae grow in the liver as a result of the invasion of the surrounding tissues, and are more hazardous than cystic echinococcosis (CE). If left untreated, the mortality rates of AE patients can be as high as 50%-75%[1,4,5]. Diagnosis of AE is supported by the results from imaging studies, histopathology, nucleic acid detection and serology [1,6].

AE is epidemic in the northern hemisphere including central Europe, most area of Russia, western Alaska, the northwestern portion of Canada, the Central Asian republics, northern Japan, and western China [1]. The dynamics of *E*. *multilocularis* transmission are complex. The reported definitive hosts include foxes and domestic dogs, wolves and cats [1,7,8]. The main intermediate hosts of *E*. *multilocularis* are small mammals [5]. People are usually infected with *E*. *multilocularis* by handling infected hosts, or by ingestion of food contaminated with eggs. AE patients are termination of *E*. *multilocularis* life cycle, because they are not generally consumed by definitive hosts and protoscolices are rarely observed in alveolar hydatid cyst in patients lesion organ [1].

Regional distributions of AE in China are limited to northwest and northern China including Xinjiang, Gansu, Ningxia, Tibet, Sichuan, Qinghai, Inner Mongolia, and Heilongjiang Provinces or Autonomous Regions [9]. Human AE is most epidemic in Yili Kazakh Autonomous Prefecture of northwest and western areas of Xinjiang, Xiji, Haiyuan and Guyuan Counties of south Ningxia, Ganze and Aba Tibetan Autonomous Prefectures of the eastern Tibetan plateau comprising northwest Sichuan, Yushu and Huangnan Tibetan Autonomous Prefectures of southwest Qinghai, Zhang and Min Counties of south Gansu [9–11].

The reported AE cases in Gansu Province were all concentrated in Zhang and Min Counties located in southern Gansu. AE in Gansu Province was first described in 1981 [12] and it was confirmed later by seroepidemiology and abdominal ultrasound scan [7]. Detailed studies were carried out in the epidemic area between 1994 and 1997 [13]. Eighty-four AE cases (3%) were identified from 2482 people in Zhang and Min Counties. Risk factors for human AE are poorly understood. Dogs played an important role in AE transmission to humans in this area. The total number of dogs owned over a period of time was shown to be a risk factor [9,13]. The proportion of habitats favorable to the vole (*Microtus limnophilus*) closely correlated to village AE prevalence rates [14]. Deforestation driven by agriculture results in creation of optimal peri-domestic habitats for rodents and subsequent development of a peri-domestic cycle involving dogs [13].

Minle County located in middle of Hexi Corridor, north slope of the Qilian Mountain, west of Gansu Province, China. Zhao et al surveyed the Minle County population hydatid diseases from 8 towns and the results showed that the prevalence was 0.75% (67/8932) in 2010 [15]. All of the 67 hydatid cases were CE, and AE cases were not found [15]. From the Zhangye People’s Hospital, our collaborators found two AE cases who lived in Nanfeng Town of Minle County in their daily health service. The current collaborative research was undertaken in this area, aiming to quantify human AE prevalence, elucidate the main risk factors and attempt to dissect the transmission ecology.

## Materials and Methods

The human study was reviewed and approved by the Ethical Review Board of School of Basic Medical Sciences, Lanzhou University. All participants and guardians provided their written consent to participate in this study. The ethics committees approved this consent procedure.

### Study area and communities

**Fig 1:**
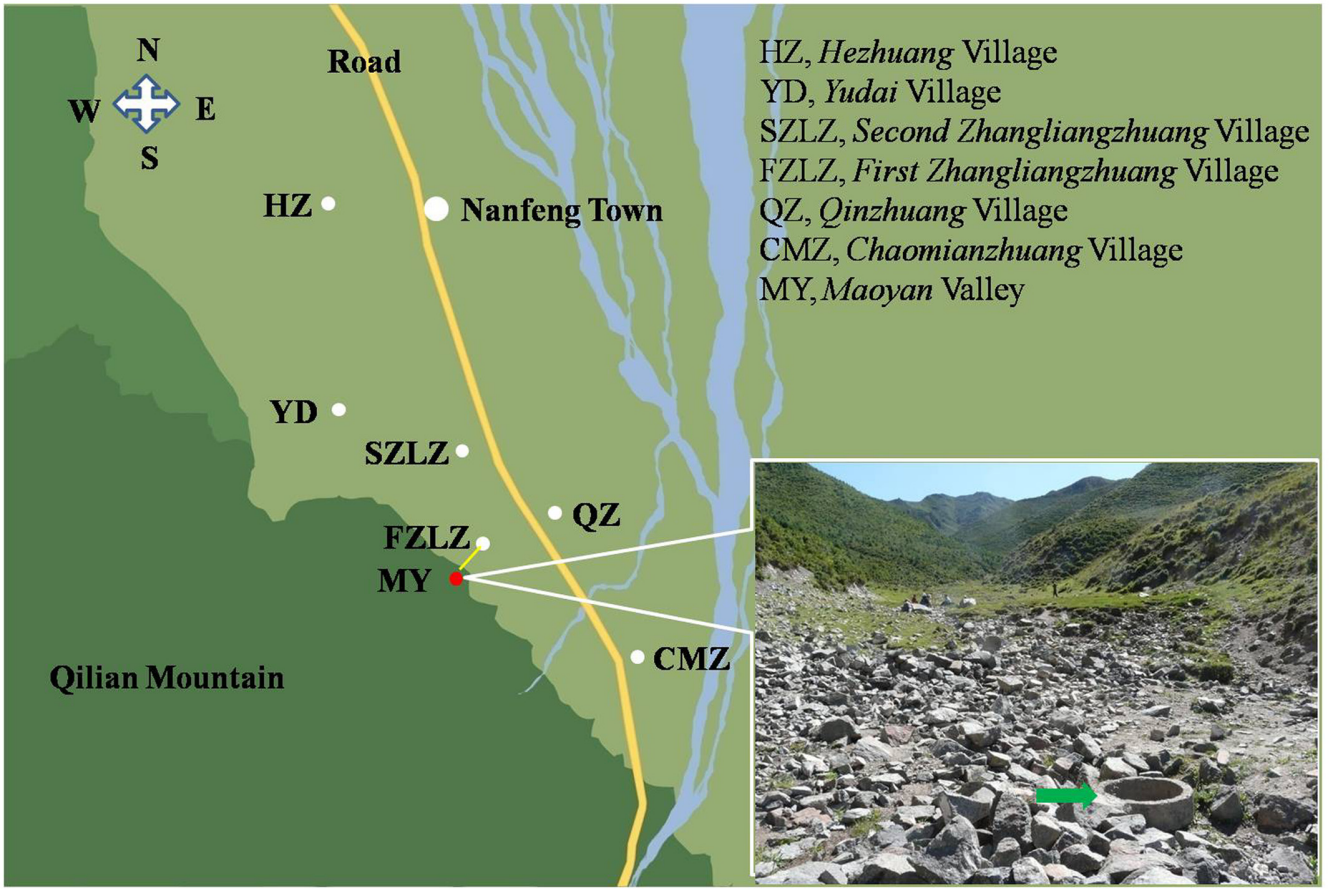
The location of FZLZ and the other five villages of Nanfeng Town of South Minle County.

The study was undertaken in six villages including *First Zhanglianzhuang* (FZLZ), *Second Zhanglianzhuang* (SZLZ), *Hezhuang* (HZ), *Qinzhuang* (QZ), *Yudai* (YD), and *Chaomianzhuang* (CMZ) which lie in Nanfeng Town of south Minle County (Fig 1), about 900 kilometers away from Zhang County of south Gansu Province. Through the majestic Qilian Mountain, these villages neighbor with Qilian County of Qinghai Province. The villages ranged in size from 256 to 2650 people with an average population of 1125. All villagers in the six villages were Han Chinese, most of them were subsistence farmers (wheat, barley, rape, potatoes, highland barley, flax, beans, etc.). Every village owns at least one infirmary, and the nearest hospital is in the Nanfeng Town with the distance to the village ranging from 1.5 to 9 km. The maximum distance between the surveyed villages is about 10 km. Livestock comprised sheep, goats, pigs, chickens, yaks and cattle. Domestic dogs were owned in most families. The whole Minle County annual average temperature is about 4.1°C. The annual average rainfall is 351 mm, with a frost-free period of approximately 140 days. The average altitude of the six villages is about 2600 m. The summer of the area is short, warm and a little dry (14–32°C) and the winter is always cold (−8–−28°C).

### Alveococcosis cases diagnosis methods

The hydatid cases medical record databases were searched from the hospitals where they were cared before. AE cases were diagnosed by performing or re-analyzing serodiagnosis, histopathology, computed tomography (CT), B-ultrasound, immunohistochemistry, DNA PCR and sequence technique. The medical record databases of CT images, pathological slice, and B-ultrasound images were examined by the experienced radiographic physicians, pathologists, and sonographers. Paraffin-embedded sample slices were stained by immunohistochemical method with polyclonal antibodies of mice infected by *E*. *multilocularis*. DNA was extracted from paraffin-embedded samples by using the special kit (Takara Bio.). *E*. *multilocularis* mitochondrial NADH dehydrogenase 1 gene was used as the target gene to design the primer based on the reference [16]. The primer EM29F (5’-GATTTGCTGATTTGTTAAAGTTAGTGATC-3’) and EM281R (5’-AGAACTTAAAAACGAATATTTATTGTAACT-3’) were synthesized by Sangon Biotech (Shanghai). The PCR products were sequenced by Beijing Genomics Institute. The sequence alignment was performed by NCBI Blastn.

### Human hydatid disease screening and questionnaires

A total of 362 people were voluntarily sampled from six villages which represented approximately 5.36% (362/6749) of the population. A liver scan was performed on each person by an experienced sonographer using a portable scanner (ALOKA-SSC-210) with a 3.0~3.5 MHz transducer. We designed a questionnaire form for registering individuals information including name, age, sex and any previous history of hydatid disease records (usually surgical) and recording information that was relevant to identifying AE risk factors which included fox hunting, fox-skins contact history, dog ownership (number and length of time), dog faeces used as fertilizer or exposure history, feeding dogs with animal organs, eating wild fruit or vegetables, a previous history of AE and living in hydatid disease epidemic areas. A 5-ml venous blood sample was taken from some people with characteristic or query echinococcosis or alveococcosis images or with hydatid disease surgical history. The seral antibody was detected by colloidal gold rapid diagnostic kit as described by the manufacturer (Xinjiang Beithming Biotechnology Development Co., LTD).

### Hosts investigation

All the investigated dogs were domestic and we obtained permission from dog owners to use the faeces. The Kato-katz technique [17] was used to detect the tapeworm eggs from 356 dogs. We could not obtain samples from other possible definitive and intermediate hosts of *E*. *multilocularis* other than dogs and instead only used pre-existing data supplied by the local Forestry Administration (the wild animal management department) and the Centers for Disease Control and Prevention of Minle County. These data were not published before.

### Statistical analysis

SPSS 17.0 was used to analyze questionnaire data for statistical significance of putative human AE ‘risk factors’ (Chi-Square-test).

## Results

### Nine AE cases and one CE case were found in the area

**Fig 2:**
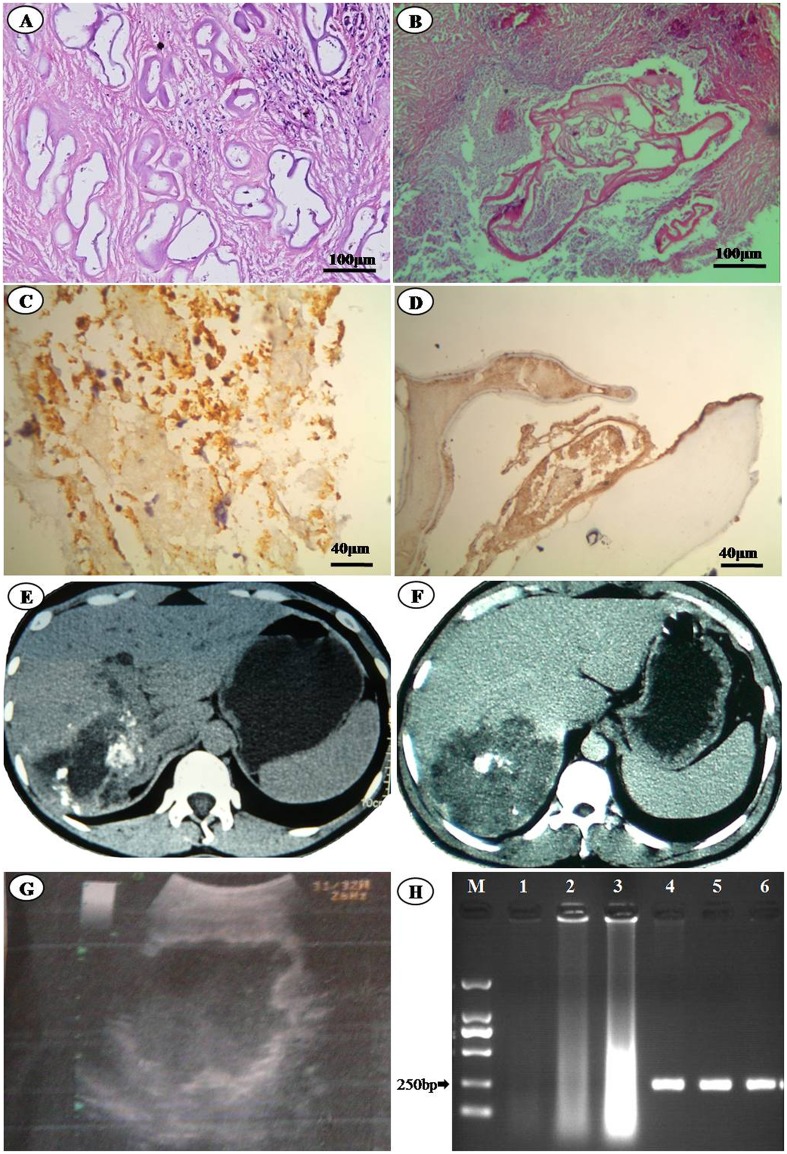
The methods used in AE diagnosis. (A) and (B) the 5^th^ and 4^th^ cases’ liver samples histopathological images (HE staining, ×100); (C) and (D) the 7^th^ and 8^th^ cases’ liver samples immunohistochemical staining images (×400); (E) and (F) the 7^th^ and 8^th^ cases’ abdominal CT images; (G) the 1^st^ cases’ abdominal B-ultrasound images; (H) DNA extracted from three cases’ paraffin- embedded samples and PCR products (M.5000 bp DNA Marker. Line 1, 2, 3, DNA extracted from the 5^th^, 7^th^, and 8^th^ cases paraffin-embedded samples. Line 4, 5, 6, PCR products of the 5^th^, 7^th^, and 8^th^ cases’ DNA samples).

Eight cases who had been diagnosed as hydatidosis in the local hospitals and treated by surgeries before were found by our questionnaire. After we re-analyzed the eight cases medical record databases, and with the addition of serodiagnosis, immunohistochemistry, DNA PCR and sequence technique, we confirmed that all the eight cases were infected by *E*. *multilocularis*. Two new hydatid cases were discovered by our B-ultrasound screen from 362 volunteers. One case image suggested AE diagnosis, and the serum *E*. *multilocularis* antibody was also positive. Another case image suggested CE diagnosis, and the serum *E*. *granulosus* antibody was positive, but the *E*. *multilocularis* antibody was negative. Thus, we found nine AE cases and one CE case in the area. The diagnostic methods and results for the 9 AE cases are listed in Table 1 and Fig 2.

**Table 1:** The diagnostic methods for the 9 AE cases.

Cases	diagnostic methods					
	Sero- diagnosis	Histopath-ology	CT	B-ultrasound	Immunohisto- chemistry	DNA PCR and sequence
1^st^ Case	+	N	N	+	N	N
2^nd^ Case	+	N	N	N	N	N
3^rd^ Case	+	+	+	+	+	+
4^th^ Case	+	+	+	+	N	+
5^th^ Case	+	+	+	+	+	+
6^th^ Case	+	N	N	N	N	N
7^th^ Case	+	+	+	+	+	+
8^th^ Case	+	+	+	+	+	+
9^th^ Case	N	N	+	+	N	N

N, not done. +, the results support alveococcosis diagnosis.

The serodiagnostic detections of eight cases (except for 9^th^ case which patient was dead) alive at that time were all positive. HE stained histopathological slices from lesions showed the characteristics of alveolar hydatid cyst (Fig 2A and 2B). The case pathological specimens immunohistochemically stained with alveococcosis mice polyclonal antibodies showed positive results (Fig 2C and 2D). CT and B-ultrasound images of the cases all supported liver AE diagnosis (Fig 2E, 2F and 2G). The PCR products size of *E*. *multilocularis* mitochondrial NADH dehydrogenase 1 gene from the paraffin-embedded samples templates was same as initial designed (252 bp) (Fig 2H). The sequence alignment showed 100% similarity with the mitochondrial NADH dehydrogenase 1 gene of 18 *E*. *multilocularis* strains in NCBI.

### The cases’ distributions and the villagers domestic water investigation

Out of nine AE cases, 8 came from FZLZ village and 1 from YD village, 2 km northwest of FZLZ village (Fig 1). All the cases were adult farmers ranging from 24 to 62 years old. The FZLZ was the nearest village to the *Qilian* Mountain among the six villages (Fig 1). Only the FZLZ villagers used the domestic water from two wells whose water came from the *Maoyan* Valley located in *Qilian* Mountain. The two wells were opened and the water from the upper spring could permeate into the well through the gravel fractures (Fig 1). Around the *Maoyan* Valley and nearby *Qilian* Mountain, the vegetation was thriving and the ground and slopes were covered by grass and *shrubs* (Fig 1). Except for FZLZ villagers, domestic water of other villagers’ came from a reservoir.

### Risk factors investigation

Statistical analysis of information in questionnaires were carried out in association with the risk factors of hydatid disease from 730 villagers (10.82% of the population) in the six villages (270 males and 460 females). 113 respondents came from FZLZ village and 617 came from other five villages. The mean age was 44.29 (ranging from 11 to 78 years). There were no significant differences detected between FZLZ villagers and other villagers in habits linked with hydatid disease (*P* value > 0.05), except that the numbers of relatives with hydatid history of the inquired FZLZ villagers were significantly higher than other villagers (*P* value < 0.05) (Table 2).

**Table 2:** Potential risk factors for human hydatids disease in Nanfeng Town. People habits were compared between FZLZ and other villages using Chi-Square-test.

Potential risk factor	FZLZ %	Other villages	*P* value	Total
Farmers	84.07 (95/113)	85.25 (526/617)	0.75	85.07 (621/730)
Fox hunting	2.65 (3/113)	2.76 (17/617)	0.95	2.74 (20/730)
Contact fox skins	5.31 (6/113)	2.92 (18/617)	0.19	3.29 (24/730)
Wild vegetables	41.59 (47/113)	32.25 (199/617)	0.053	33.70 (246/730)
Vegetable garden	69.91 (79/113)	69.69 (430/617)	0.84	69.73 (509/730)
Dog ownership	67.26 (76/113)	61.43 (379/617)	0.24	62.33 (455/730)
Dog faeces as fertilizer	12.39 (14/113)	18.96 (117/617)	0.09	17.95 (131/730)
Sheep ownership	69.03 (78/113)	70.02 (432/617)	0.83	69.86 (510/730)
Sheep liver feed dog	14.16 (16/113)	9.24 (57/617)	0.11	10.0 (73/730)
Relatives with hydatid history	23.89 (27/113)	6.32 (39/617)	<0.05	9.04 (66/730)

### Possible AE hosts investigation

No tapeworm egg was found from 356 dogs’ faeces by using Kato-katz technique. The geographic diversity in Minle County provides good natural conditions for wildlife and the area has a variety of wild species including about 58 kinds of mammals species, 142 kinds of birds species and 13 kinds of amphibians and reptiles species. Besides the dogs, other possible definitive hosts of *E*. *granulosus* and *E*. *multilocularis* are *Vulpes vulpes*, *Canis lupus*, *Cuon alpinus*. Many villagers had occasionally seen the red fox in nearby areas in winter. Rodents are the primary intermediate hosts of *E*. *multilocularis*[1]. There are several rodents including *Ochotona thomasi*, *Mus musculus*, *Rattus norvegicus*, *Cricetulus migratorius*, *Microtus irene*, *Microtus oeconomus*, *Marmota himalayana* in the area. The dominant species of wild rodents is *O*. *thomasi* and the dominant species in residential areas is *M*. *musculus*. The other wild herbivores of *Lepus oiostolus* and *Lepus capensis* in the area can also serve as possible intermediate hosts of *E*. *multilocularis* [18–23].

## Discussion

### Nanfeng Town of Minle County in west Gansu Province is a newly discovered area of *E*. *multilocularis* prevalence in China

**Fig 3:**
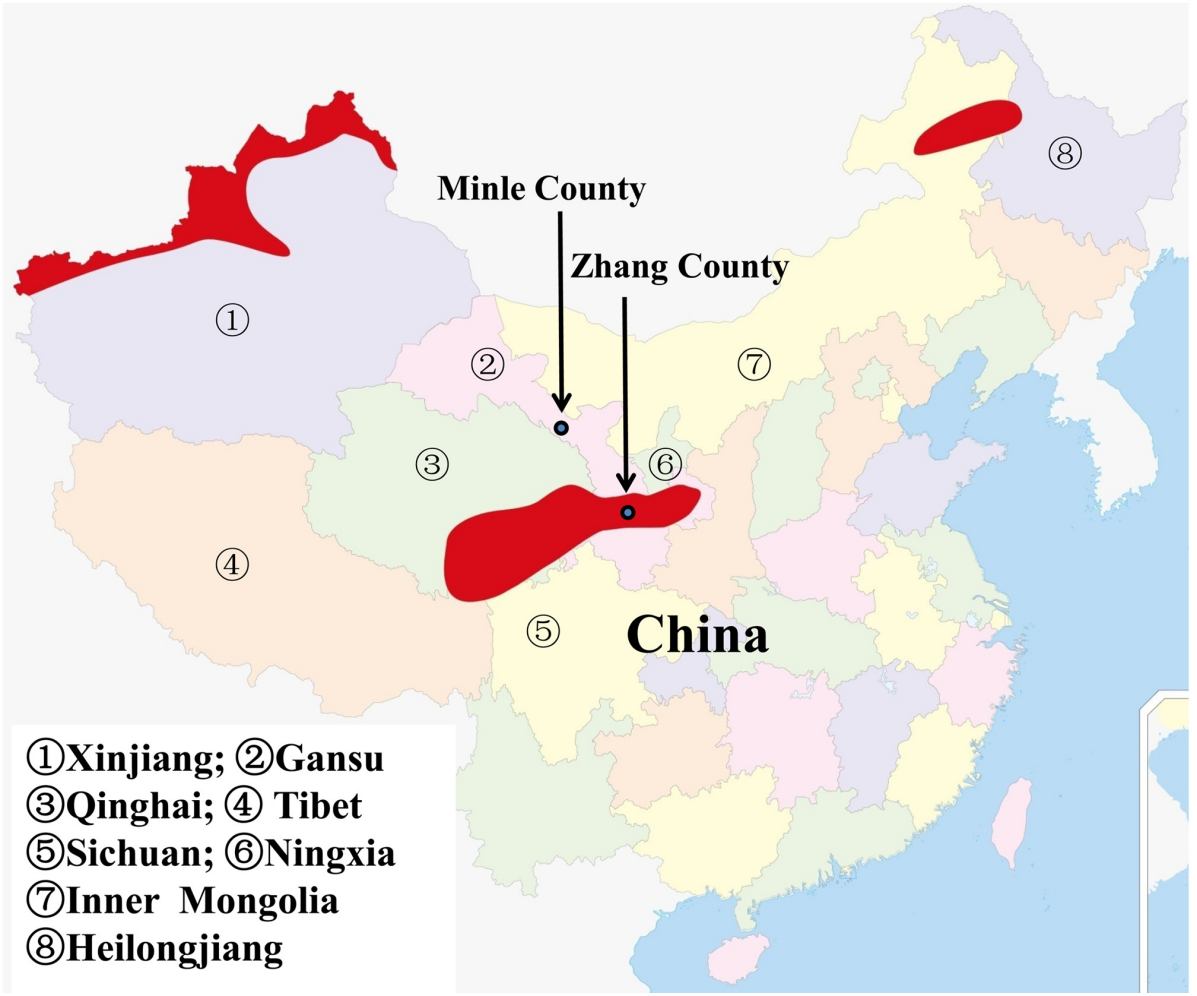
Epidemiology of human AE in China, and the location of Minle County and Zhang County in China. The major epidemic regions of human AE in China (the red areas). Minle County locates in west Gansu Province and about 900 km away from Zhang County, and outside of the red areas. This figure is similar but not identical to the image which was published by Craig [9].

The previous known areas of AE in Gansu Province were Zhang and Min Counties [7]. In this study, we provide evidence of nine AE cases in Nanfeng Town of Minle County. Except for one case dwelling in YD village, other eight cases lived in FZLZ village. The questionnaires showed that all the cases had never visited the historical foci of Zhang-Min County before. Moreover, Nanfeng Town is situated in the skirt of the Qilian Mountain and close to Tibetan plateau foci of Qinghai. Its geographical location, the altitude and cold climatic conditions make the area suitable for *E*. *multilocularis* endemicity. Nanfeng Town of Minle County is a newly discovered focus of AE in China (Fig 3).

### The local AE transmission dynamics analysis

The transmission pattern of *E*. *multilocularis* includes both the sylvatic cycle and the synanthropic cycle [11]. The sylvatic cycle is the main transmission pattern of *E*. *multilocularis* in Europe, Japan, and North America [24,25]. However, in China, epidemiological studies generally indicated that the synanthropic cycle, involving dogs, seemed to be the main transmission route to humans [8,9,11,26]. In this study, we did not detect significant differences regarding dog ownership between the participants screened in FZLZ, and those of other villages, and did not found taeniid egg in 356 dogs’ faeces. Furthermore, we found that only the domestic water of the FZLZ was different from other villages. The government has established wildlife sanctuaries in those mountains, which provides good natural conditions for wildlife. The data, provided by the local Forestry Administration and the Centers for Disease Control and Prevention of Minle County, indicated that besides domestic dogs, three species of wild animals could act as the possible definitive hosts and other nine species animals could be the possible intermediate hosts. We speculate that red foxes (*V*. *vulpes*) and wolves (*C*. *lupus*), and the variety of small mammals, which thrive in the mountains which may favor a sylvatic cycle of *E*. *multilocularis* transmission possibly, and it needs a further confirmation. The vegetation of grass and *shrubs* around the *Maoyan* Valley and nearby *Qilian* Mountain provides a suitable habitat for wildlife especially foxes (Fig 1). Considering the facts that the distance of other five villages to the FZLZ village are very near, and all natural conditions are similar except the domestic water source, and the morbidity of AE is so different, the possible explanation is that the definitive host (such as foxes etc.) faeces in the *Maoyan* Valley can be washed into the well possibly and be a source of *E*. *multilocularis* infection.

### Minle County is a CE and AE co-epidemic area

In most places, human CE and AE are epidemic separately, but they are co-epidemic in only a few other regions, notably eastern Turkey, central Asia and Siberia [27]. The majority of echinococcosis cases in China are caused by *E*. *granulosus*[28], but in some areas, CE and AE exist in a co-epidemic phenomenon, and these areas include Qinghai, Sichuan and Gansu Provinces and Xin Jiang and Ningxia Autonomous Regions [28–30]. The communities where CE and AE are co-epidemic always suffered from serious echinococcosis, and dogs always were important sources for both *E*. *granulosus* and *E*. *multilocularis*[11,28]. However in Nanfeng Town, we did not detect that dogs are source for *E*. *multilocularis* during the course of our study.

Other investigators reported that the CE occurred in Nanfeng Town with a prevalence rate up to 1.5%[15]. In this study, we found one CE case and nine AE cases in Nanfeng Town. Our finding suggests that Nanfeng Town of Minle County is a CE and AE co-epidemic area and that the area is an important echinococcosis epidemic area. Although we could not confirm that dogs played a role in AE propagation in the local area, other geographical results warned us that dogs in Nanfeng Town should be monitored for *E*. *multilocularis* infection in order to prevent AE prevalence.

FZLZ village is centralization cluster of AE cases. The village is situated at the northern foot of Qilian Mountain. Across the *Qilian* Mountain, the *E*. *multilocularis* can be transmitted to the Qinghai Province, and along the *Qilian* Mountain it can be transmitted to other places of the vast Hexi Corridor. It is important to carry out further research to explore the *E*. *multilocularis* transmission pattern in order to develop strategies to prevent the parasitic disease transmitted in the area.
